# HBV-Induced Pyruvate Increases Lactylation of Pyruvate Kinase M2 (PKM2) at K206 to Promote Liver Fibrosis

**DOI:** 10.3390/pathogens15040431

**Published:** 2026-04-16

**Authors:** Wenxian Wen, Qin Du, Shuhan Li, Youmin Yang, Xianding Wang, Shasha Li, Yujia Li, Shilin Li, Chunhui Yang, He Xie, Xiaoqiong Duan, Limin Chen

**Affiliations:** 1 Department of Clinical Medicine, North Sichuan Medical College, Nanchong 637000, China; 2 Institute of Blood Transfusion, Chinese Academy of Medical Sciences & Peking Union Medical College, Chengdu 610052, China; 3 Department of Urology, Institute of Urology, Kidney Transplant Center, West China Hospital, Sichuan University, Chengdu 610041, China; 4 Department of Hepatology, The Second People’s Hospital of Fuyang City, Fuyang 236015, China; 5 The Hospital of Xidian Group, Xi’an 710077, China; 6 The Joint Laboratory on Transfusion-Transmitted Diseases (TTDs) Between Institute of Blood Transfusion, Chinese Academy of Medical Sciences and Nanning Blood Center, Nanning Blood Center, Nanning 530007, China

**Keywords:** hepatitis B virus, pyruvate, liver fibrosis, PKM2, lactylation

## Abstract

We previously demonstrated that HBV promotes liver fibrosis through the enhanced production of pyruvate. Pyruvate kinase M2 (PKM2), a key enzyme in pyruvate metabolism, plays an important role in liver fibrogenesis. Recently, lactylation of PKM2 has been identified, which contributes to stabilize its catalytically active tetrameric conformation. Therefore, we hypothesize that PKM2 lactylation is involved in the regulation of HBV-induced liver fibrosis. In this study, we found that sera lactate levels were increased in CHB patients and HBV-Tg mice. Moreover, the lysine lactylation levels of proteins in liver tissues were significantly increased in the HBV-Tg mice. In LX2 cells, we found that pyruvate treatment significantly increased the profibrotic gene expression and lactylation level of PKM2, which promoted its tetramer-to-dimer transition, inhibited its pyruvate kinase activity, and facilitated its nuclear distribution. Through immunoprecipitation, we identified that pyruvate induced PKM2 lactylation at the K206 site. PKM2 knockdown or K206 mutation reduced PKM2 lactylation and abrogated the induction of profibrotic gene expression by pyruvate. Collectively, our findings indicate that HBV infection stimulated pyruvate production, which increased PKM2 lactylation at K206 to promote the expression of profibrogenic genes in HSCs, leading to liver fibrogenesis.

## 1. Introduction

HBV infection remains a major public health issue globally. Approximately 296 million people worldwide are hepatitis B surface antigen (HBsAg)-positive carriers, with 1.5 million new cases of HBV infection reported annually and around 820,000 deaths reported annually due to HBV-related complications [1]. Chronic hepatitis B (CHB) leads to severe hepatic complications including liver fibrosis, cirrhosis, and hepatocellular carcinoma (HCC) [2]. Liver fibrosis is a chronic liver disease caused by repeated and long-term parenchymal injury from diverse etiologies including alcohol, hepatic toxins, chronic infections, autoimmune disorders, and metabolic dysregulation [3,4,5]. During liver injury, quiescent hepatic stellate cells (HSCs) are activated and differentiate into myofibroblast-like cells, accompanied by the production of a large amount of collagen 1α1 (COL1A1) and tissue inhibitors of metalloproteinases (TIMPs), leading to the development of liver fibrosis [6]. Although antiviral therapy can effectively suppress viral replication and partially reverses liver fibrosis in some CHB patients, accumulating evidence suggests that disease progression is also closely linked to virus-induced host metabolic abnormalities [7,8,9].

It has been reported that HBV infection upregulates the expression of critical glycolytic enzymes in host cells to activate glycolysis, resulting in the accumulation of metabolites including pyruvate, phosphoenolpyruvate, and lactate [10,11]. We previously reported that pyruvate promoted HBV replication in hepatocytes [12] and increased profibrotic gene expression through increasing the production of reactive oxygen species (ROS) [13]. As a key intermediate in cellular metabolism, pyruvate can either be converted to acetyl-CoA under aerobic conditions or lactate under anaerobic conditions [14]. Lactylation is a newly discovered protein post-translational modification (PTM) driven by lactate [15] and has been reported to be involved in the progression of liver fibrosis [16]. For example, Rho et al. reported that lactate-mediated H3K18 lactylation promoted the activation of HSCs [17]. However, the association and interplay of HBV-induced pyruvate, lactylation, and liver fibrosis are still unknown.

Pyruvate kinase (PK), a key rate-limiting enzyme of glycolysis, plays a critical role in pyruvate metabolism. There are four isozymes (M1, M2, R, L) of PK in mammals, and PKM2 is specifically generated through the alternative splicing of PKM gene transcripts [18]. PKM2 exists in two oligomeric states: a tetramer with high PK activity and a dimer with low PK activity. The tetrameric form catalyzes the conversion of phosphoenolpyruvate (PEP) to pyruvate in cytoplasm, whereas the dimeric form translocates into the nucleus to regulate gene transcription by activating specific transcription factors such as c-Myc, β-catenin, and cyclin D1 (CCND1) [19,20,21,22,23,24,25]. Some glycolysis-related enzymes have been found to be regulated by dimeric PKM2, which promotes aerobic glycolysis in most cancer cells [26]. It has also been reported that PKM2 is abundantly expressed in a low-activity dimeric form in activated HSCs, where it enhances aerobic glycolysis to accumulate intermediates that provide energy and metabolic precursors for collagen synthesis, thereby directly accelerating fibrotic progression [27]. Post-translational modifications are known to affect the function and allosteric regulation of PKM2 [28]. Wang et al. reported that lactate treatment significantly increased PKM2 lactylation at the K62 site in macrophages, which inhibited its tetramer-to-dimer transition and consequently promoted the shift of pro-inflammatory macrophages to a reparative phenotype [29]. However, whether PKM2 lactylation is involved in the accumulation of dimer PKM2 in activated HSCs or plays a role in HBV-induced liver fibrosis remains unclear. In this study, we demonstrated that HBV-induced pyruvate production increased PKM2 lactylation at K206, which upregulates the expression of profibrogenic genes in HSCs and promotes the progression of liver fibrosis.

## 2. Materials and Methods

### 2.1. Cells and Reagents

LX2, NTCP-HepG2, and HEK293T cells were routinely maintained in our laboratory and were cultured in DMEM (Gibco, Carlsbad, CA, USA) supplemented with 10% fetal bovine serum (Biological Industries, Kibbutz Beit Haemek, Israel) and 1% mycoplasma prevention reagent (TransGen Biotech, Beijing, China) at 37 °C in a 5% humidified CO_2_ incubator. Cells were seeded in 6-well plates at a density of 3 × 10^5^ cells per well without sodium pyruvate overnight and then treated with or without sodium pyruvate for 12 h.

### 2.2. siRNAs, Plasmids, and Transfection

PKM2 siRNA and corresponding negative controls (Neg siRNA) were purchased from Sangon Biotech (Shanghai, China) and transfected into cells using the Rfect V2 siRNA Transfection Reagent (Baidai biotechnology, Changzhou, China). pPKM2-Flag-WT and the corresponding empty vector control (pEmpty) were purchased from MIAOLING BIOLOGY (Wuhan, China). The pPKM2-Flag-WT plasmid was amplified using KOD OneTM PCR Master Mix-Blue (Toyobo, Osaka, Japan) with complementary primers containing the target mutation sites. After PCR amplification, the original methylated template plasmid was digested with DMT enzyme (TransGen Biotech, Beijing, China). The purified PCR products were directly transformed into DMT-competent cells (TransGen Biotech, Beijing, China), and the mutation sites were subsequently verified by sequencing to obtain Flag-tagged PKM2 mutants. Plasmid DNA was transfected into cells using PEI MAX^®^ (Polysciences, Warrington, PA, USA) according to the manufacturer’s instructions.

### 2.3. RNA Isolation, Reverse Transcription, and RT-qPCR

Total RNA was extracted using Trizol reagent (Invitrogen, Carlsbad, CA, USA). RNA was reverse-transcribed to cDNA using ReverTra Ace^®^ qPCR RT Master Mix with gRNA remover (Toyobo, Osaka, Japan). The cDNA products were diluted with nuclease-free water for gene detection by quantitative PCR (qPCR) with NovoStart^®^ SYBR qPCR SuperMix Plus Kits (Novoprotein, Suzhou, China). mRNA levels of selected genes were calculated using the 2^−∆∆Ct^ method and normalized to *β-actin* as the housekeeping gene internal control. Primers for RT-qPCR are listed in Table 1.

### 2.4. Western Blot

Protein samples were prepared from cell lysates using RIPA lysate (Beyotime, Shanghai, China) containing protease inhibitor (Solarbio, Shanghai, China) and Trichostatin A (TSA) (TargetMol, Shanghai, China). Protein concentration was determined by the BCA method. A total of 20 μg proteins were separated by SDS-PAGE and transferred to an Immobilon^®^-P membrane (Millipore, Billerica, MA, USA). The primary antibodies used in this study included anti-PKM2, anti-IgG, anti-β-actin, anti-FLAG, anti-TIMP1 (Proteintech, Wuhan, China), anti-COL1A1 (ABclonal, Wuhan, China), and anti-L-lactyl lysine (PTM Bio Inc., Hangzhou, China). The secondary antibodies include HRP-conjugated goat anti-mouse IgG (H + L) and HRP-conjugated goat anti-rabbit IgG (H + L) (Proteintech, Wuhan, China). The chemiluminescence assay was performed using the Immobilon^®^ Western Chemiluminescent HRP Substrate (Millipore, USA) in ChemiDoc MP 9 (Bio-Rad, Hercules, CA, USA). Densitometry analysis of Western blot protein bands was performed with ImageJ 1.54p software. β-Actin was used as a loading control, and protein expression was normalized to that of β-actin.

### 2.5. Immunoprecipitation (IP)

Cells were lysed in RIPA lysis buffer (Beyotime, Shanghai, China). After centrifugation, the supernatant was incubated with specific antibody and protein A/G beads (Proteintech, Wuhan, China) or anti-DYKDDDDK magnetic beads (GenScript, Nanjing, China) at 4 °C overnight. The bead mixture was washed three times with washing buffer and boiled at 95 °C in SDS loading buffer for 5 min. Finally, proteins bound to the beads were analyzed by Western blot as described above.

### 2.6. Cell Viability Assay

The viability of the LX2 and NTCP-HepG2 cells was measured by a Cell Counting Kit 8. Briefly, cells were seeded in 96-well plates at a density of 2000 cells per well. A total of 10 μL CCK8 (Biosharp, Hefei, China) was added to each well, followed by incubation for 2 h at 37 °C. The absorbance value at 450 nm was measured using a microplate reader.

### 2.7. PKM2 Crosslinking

Cells were collected and washed three times with ice-cold PBS and then incubated in PBS containing 500 μM disuccinimidyl suberate (DSS; Thermo Fisher, Waltham, MA, USA) for 30 min at room temperature. A total of 10 mM Tris (pH 8.0) was added into cell suspension at room temperature for 15 min to terminate the crosslinking before the cells were collected and lysed in RIPA buffer for Western blot analysis.

### 2.8. Nuclear and Cytoplasmic Protein Extraction

Cells were collected and incubated in a hypotonic buffer (20 mM Tris-HCl, pH 7.4, 10 mM NaCl, 3 mM MgCl_2_) on ice for 15 min. NP-40 (Sigma, St. Louis, MO, USA) was added and vortexed for 10 s. The supernatant was collected after centrifuging at 2000× *g* for 4 °C for the detection of cytoplasmic protein. The remaining pellet was resuspended with 100 uL RIPA lysis buffer and lysed on ice for 10 min to ensure thorough nuclear membrane disruption. Following spinning down at 20,000× *g* for 4 °C, the supernatant was collected for nuclear protein detection. PKM2 levels in the cell cytoplasm and nucleus were detected separately by Western blot.

### 2.9. Measurement of Lactate and Pyruvate Kinase Activity

Levels of serum lactate from human and mice were assessed using a Lactic Acid (LA) Content Assay Kit (Solarbio, Shanghai, China) following the manufacturer’s protocol. The pyruvate kinase (PK) activity was examined using a Pyruvate Kinase (PK) Activity Assay Kit (Solarbio, Shanghai, China) according to the manufacturer’s recommended procedures.

### 2.10. Human Subject Study Ethics Approval and Animal Study Approval

All of the human subject study and animal study protocols were approved by the Ethics Committee of the Institute of Blood Transfusion, Chinese Academy of Medical Sciences and Peking Union Medical College (No. 2021020, 2024004). This study complied with the ethical standards of the institutional and national research committee and adhered to the principles outlined in the Declaration of Helsinki of 1964. Sera from patients and healthy controls, and the corresponding clinical data, were collected from the Department of Hepatology, the Second People’s Hospital of Fuyang City, as previously reported [13]. The clinical data of all patients are presented in Table 2. The serum study protocol was approved by the Ethics Review Committee of the Second People’s Hospital of Fuyang City (approval number: 20210901014).

HBV transgenic male mice with a C57BL/6N background carrying a 1.28-fold overlength HBV genome (genotype A) were purchased from Beijing Vitalstar Biotechnology Ltd., (Beijing, China), and wild-type (WT) C57BL/6N male mice were used as controls. The experimental protocol was approved by Vitalstar IACUC (No. VST-SY-202407301).

### 2.11. Statistical Analysis

All experiments were performed at least three times independently, and the data are presented as the mean ± SD (cell-based experiments) or mean ± SEM (human and mouse experiments). In the statistical analysis, comparisons between two groups were conducted using the two-tailed Student’s *t*-test, while comparisons among multiple groups utilized one-way ANOVA. The significance level was set at *p* < 0.05, with statistical symbols denoted as follows: *p* < 0.05 (*), *p* < 0.01 (**), *p* < 0.001 (***), *p* < 0.0001 (****), and not significant (ns) for *p* > 0.05. All statistical analyses were performed using GraphPad Prism 9.0 software. Post hoc power analysis was conducted using G*Power version 3.1.9.7 to calculate the achieved power and effect size (Cohen’s d).

## 3. Results

### 3.1. HBV Infection and Pyruvate Treatment Increased Protein Lactylation

**Figure 1:**
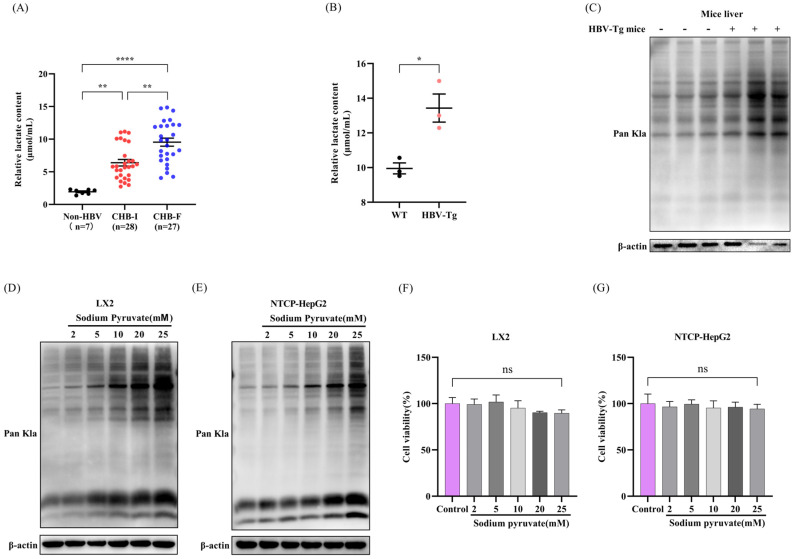
HBV induced glycolysis to promote protein lactylation. (**A**) Serum lactate levels were significantly increased in CHB patients compared to HBV-uninfected controls (Non-HBV). Additionally, the serum lactate level in the CHB-F (advanced fibrotic stage of chronic hepatitis B virus) group was higher than that in the CHB-I (inactive carrier stage of chronic hepatitis B virus) group. (**B**) Serum lactate levels were significantly increased in HBV-Tg mice compared to wild type mice (n = 3). (**C**) Protein lactylation levels significantly increased in liver tissues from HBV-Tg mice compared to the WT controls. (**D**) Pyruvate treatment markedly increased protein lactylation in LX2 cells. (**E**) Pyruvate treatment markedly increased protein lactylation in NTCP-HepG2 cells. (**F**) Cell viabilities of LX2 cells were not affected by pyruvate treatment at 12 h. (**G**) Cell viabilities of NTCP-HepG2 cells were not affected by pyruvate treatment at 12 h. Data were expressed as mean ± SD or mean ± SEM. Not significant (ns), *p* > 0.05, * *p* < 0.05, ** *p* < 0.01 and **** *p* < 0.0001.

Upon testing the serum lactate levels of the collected patient cohort, serum lactate levels were markedly elevated in HBV-infected patients compared with the HBV-uninfected controls. Moreover, among the HBV-infected patients, those with liver fibrosis displayed markedly higher lactate levels than those without fibrosis (Figure 1A). Similar findings were observed in the HBV-Tg mice compared with the wild type C57BL/6N mice (Figure 1B). These results are consistent with our and other previous findings [11,13]. Subsequently, we harvested liver proteins from both wild-type and HBV-Tg mice for lactylation profiling. We found that the liver tissues of HBV-Tg mice exhibited a significantly increased level of protein lactylation (Figure 1C). As pyruvate serves as the metabolic precursor of lactate, we therefore speculated that HBV increased protein lactylation through the increased production of pyruvate. To verify our hypothesis, we treated LX2 and NTCP-HepG2 cells with increasing concentrations of sodium pyruvate for 12 h and detected its effect on protein lactylation. We found that pyruvate significantly increased protein lactylation in a dose-dependent manner in both LX2 and NTCP-HepG2 cells without affecting cell viability (Figure 1D–G). These findings collectively suggest that HBV-induced pyruvate production enhances hepatic protein lactylation modification.

### 3.2. Pyruvate Induced Profibrotic Gene Expression in LX2 Cells Through Enhanced PKM2 Lactylation

**Figure 2:**
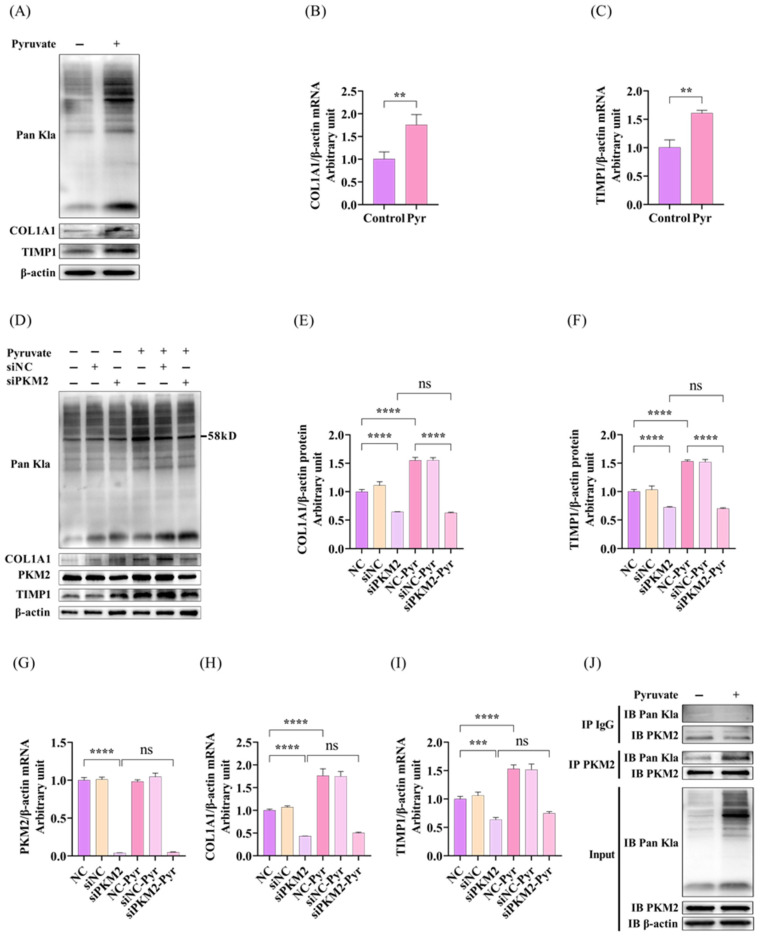
Knockdown of PKM2 reduced the induction of protein lactylation and ECM gene expression by pyruvate in LX2 cell. LX2 cells were seeded and transfected with PKM2 siRNA (45 pmol/mL) or negative control siRNA for 72 h. Cells were subsequently treated with or without 5 mM sodium pyruvate for 12 h prior to harvest. Total RNA and protein were extracted for the analysis of selected gene expression by RT-qPCR and Western blot, respectively. (**A**) Pyruvate treatment significantly increased the levels of protein lactylation and COL1A1 and TIMP1 expression in LX2 cells. (**B**,**C**) Pyruvate treatment significantly increased the mRNA levels of COL1A1 and TIMP1. (**D**–**F**) Pyruvate-induced upregulation of the COL1A1 and TIMP1 protein levels and protein lactylation. PKM2 knockdown significantly reduced the COL1A1 and TIMP1 protein levels as well as protein lactylation at about 58 kD. The relative COL1A1 and TIMP1 protein levels compared with β-actin are shown as a bar graph. (**G**) PKM2 siRNA significantly knocked-down the PKM2 mRNA level in LX2 cells. (**H**,**I**) Knockdown of PKM2 significantly decreased the mRNA levels of COL1A1 and TIMP1, and blocked pyruvate-induced COL1A1 and TIMP1 expression. (**J**) Pyruvate treatment significantly increased PKM2 lactylation. LX2 cells were treated with or without 5 mM sodium pyruvate for 12 h. The cell lysates were subjected to immunoprecipitation using PKM2-specific antibody, followed by detection via western blot using an anti-lactyllysine antibody. Data were expressed as mean ± SD of three sample replicates. Not significant (ns), ** *p* < 0.01, *** *p* < 0.001, and **** *p* < 0.0001.

To explore the effect of pyruvate on fibrogenesis, we treated LX2 cells with 5 mM sodium pyruvate and detected levels of protein lactylation and profibrotic gene expression. We observed that pyruvate treatment synchronously increased the levels of protein lactylation and profibrotic gene (COL1A1 and TIMP1) expression at both the mRNA and protein levels (Figure 2A–C). PKM2 is a key enzyme involved in pyruvate metabolism in cells, and recent studies have shown that PKM2 was markedly upregulated in HBV-infected liver tissues to promote HSC activation [13,27,30]. Therefore, we further investigated whether pyruvate-induced protein lactylation and profibrotic gene expression was associated with PKM2 regulation. We knocked-down PKM2 using specific siRNA in LX2 cells and then treated the cells with or without pyruvate. We confirmed that pyruvate treatment significantly increased the mRNA and protein levels of COL1A1 and TIMP1. Knockdown of PKM2 significantly reduced these profibrotic gene expression and mitigated the profibrotic gene induction by pyruvate treatment (Figure 2D–I). Furthermore, we observed that knockdown of PKM2 alone did not affect overall protein lactylation significantly, but attenuated the lactylation at about 58 kD in pyruvate-treated cells (Figure 2D). Considering that the molecule weight of PKM2 is around 58 kD, we speculated that pyruvate increased PKM2 lactylation. To test this, we performed PKM2 immunoprecipitation using a PKM2-specific antibody in LX2 cells and detected lactylation levels by anti-lactyllysine antibody. Our results confirmed that PKM2 lactylation was detectable and significantly enhanced following 12-h treatment with 5 mM sodium pyruvate (Figure 2J). These results collectively suggest that pyruvate induces profibrotic gene expression in LX2 cells and that this effect is mediated by enhanced PKM2 lactylation.

### 3.3. Pyruvate Promotes PKM2 Lactylation at Lysine 206

**Figure 3:**
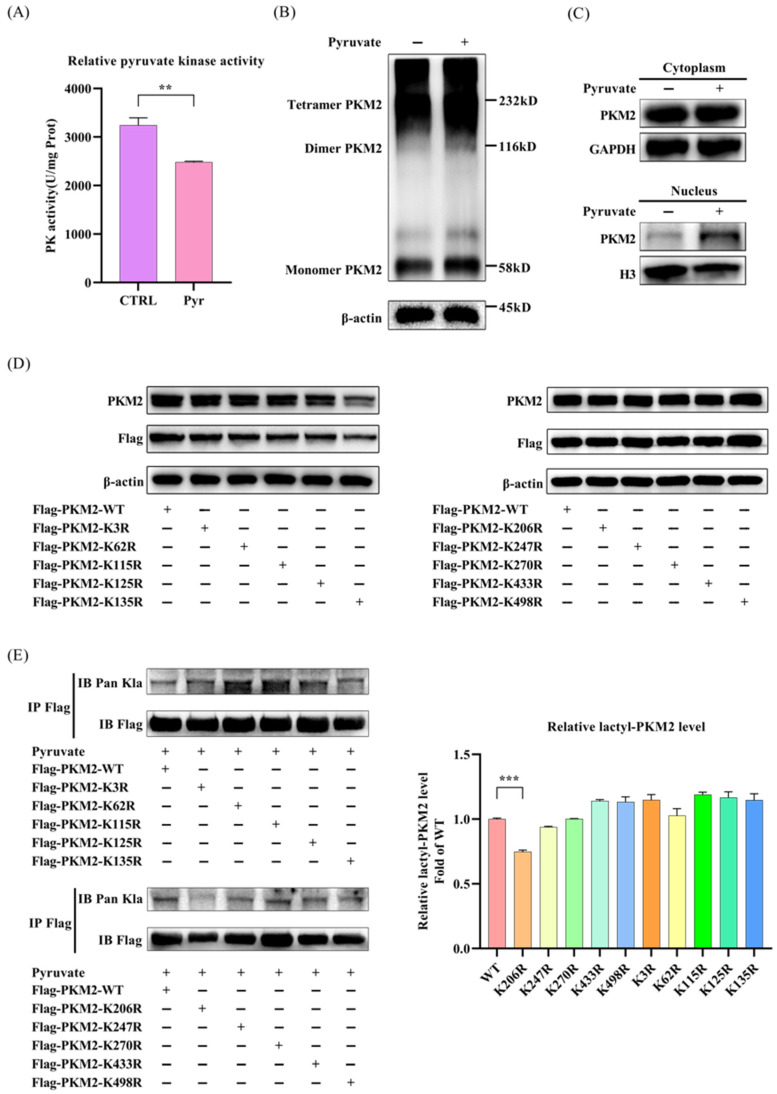
Pyruvate promotes K206 lactylation of PKM2. (**A**) Pyruvate treatment significantly reduced PKM2 activity. LX2 cells were treated with or without 5 mM sodium pyruvate for 12 h. Cell lysates were collected, and pyruvate kinase activity was detected using pyruvate kinase (PK) activity assay kits. (**B**) Pyruvate treatment significantly increased dimeric PKM2. LX2 cells were treated with or without 5 mM sodium pyruvate for 12 h, and DSS cross-linking was performed as described in the Section 2. PKM2 oligomeric states (tetramer: 232 kD, dimer: 116 kD, monomer: 58 kD) were resolved by Western blot. (**C**) The level of PKM2 in the cell nucli was significantly increased in the LX2 cells. Cytosolic and nuclear proteins were purified from LX2 cells separately, and PKM2 levels were assessed by Western blot. (**D**) WT or the mutant Flag-PKM2 plasmids were successfully expressed in cells. 293T cells were seeded and transfected with the WT or the mutant Flag-PKM2 plasmids (K3R, K62R, K115R, K125R, K135R, K206R, K247R, K270R, K433R, K498R) for 72 h, respectively. Cells were lysed and PKM2 expression was detected by Western blot. (**E**) The K206R mutant significantly attenuated pyruvate-induced PKM2 lactylation. 293T cells were transfected with the WT or the mutant Flag-PKM2 plasmids for 72 h. Sodium pyruvate (5 mM) were added 12 h before cells were lysed. Cell lysates were subjected to immunoprecipitation using anti-FLAG antibody, followed by detection via Western blot using an anti-lactyllysine antibody. The relative lactyl-PKM2 level compared with Flag-PKM2 is shown as a bar graph. Data are expressed as the mean ± SD of three sample replicates. ** *p* < 0.01 and *** *p* < 0.001.

Next, we investigated whether the lactylation of PKM2 affects its pyruvate kinase (PK) activity. We measured the kinase activity of PKM2 using a pyruvate kinase (PK) activity assay kit, and performed cross-linking experiments to detect the oligomeric state of PKM2. We found that pyruvate treatment reduced the PK activity of PKM2 by approximately 25% in LX2 cells (Figure 3A). Consistent with this, DSS cross-linking analysis of cell lysates showed an increase in dimeric PKM2 with low-PK activity (116 kDa) in the pyruvate-treated cells compared to the untreated controls (Figure 3B). As dimeric PKM2 enters the nucleus to regulate target gene expression, we further tested the PKM2 levels in the cytoplasm and the cell nucleus separately. We found that PKM2 in the nucleus was significantly increased in the pyruvate-treated LX-2 cells (Figure 3C). All of these results indicate that pyruvate reduces the PK activity of PKM2 and promotes its nuclear translocation in LX2 cells.

Recently, 16 lysine lactylation sites on PKM2 have been identified in liver tissues from HBV-associated hepatocellular carcinoma patients [31]. To confirm which lysine(s) of PKM2 was lactylated in pyruvate-treated cells, we constructed Flag-tagged PKM2 mutant plasmids with lactylation site mutations. Ten site-mutation plasmids (K3R, K62R, K115R, K125R, K135R, K206R, K247R, K270R, K433R, K498R) were successfully constructed through site-directed mutagenesis. We confirmed the expression of the PKM2 wild type (WT) and these mutant plasmids by transfecting them individually into 293T cells (Figure 3D). Then, the lactylation levels of each PKM2 mutant were assessed following immunoprecipitation with an anti-FLAG antibody. Compared with the WT PKM2, we found that K206R mutation dramatically reduced the lactylation level of PKM2, while the other nine mutants showed no significant change in lactylation levels (Figure 3E). Together, these results demonstrate that pyruvate stimulates PKM2 lactylation at lysine 206.

### 3.4. The K206R Mutation Reversed Both the Inhibition of PKM2 Kinase Activity and Blunted the Induction of Pro-Fibrotic Gene Expression Induced by Pyruvate

**Figure 4:**
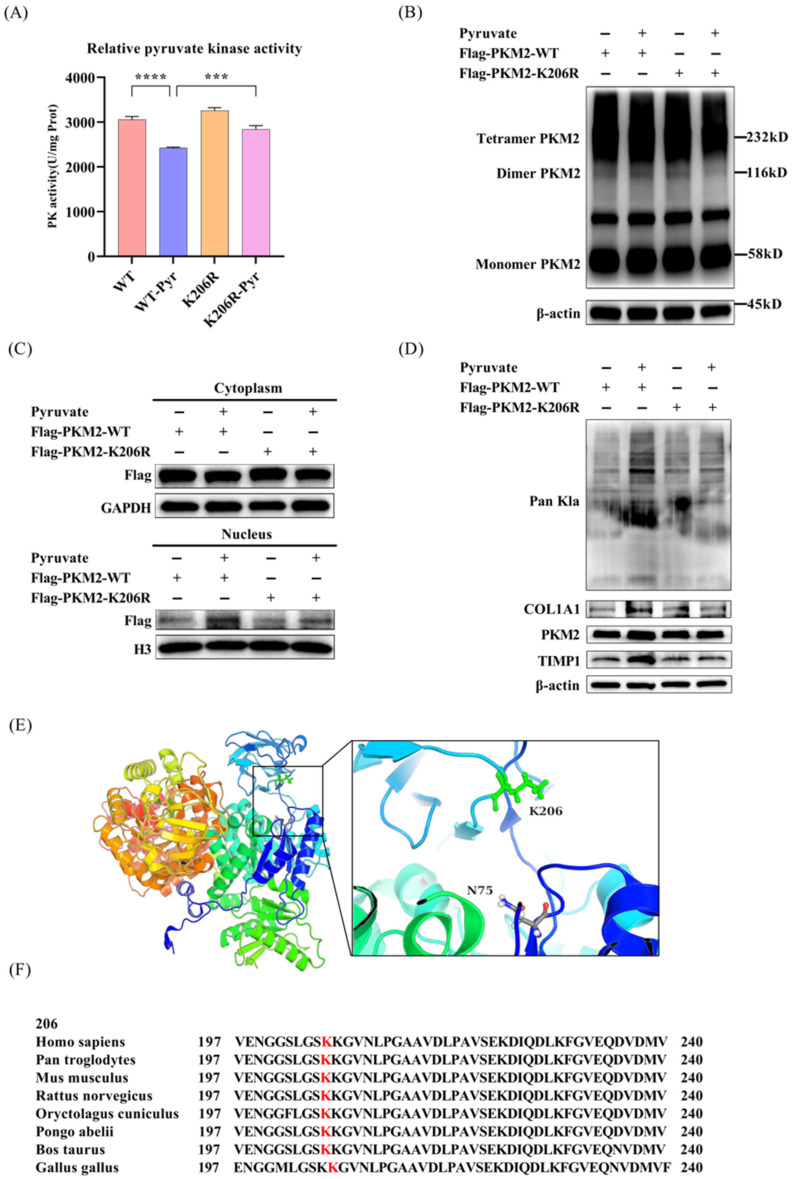
The K206R mutation reverses the pyruvate-induced suppression of PKM2 pyruvate kinase activity and pro-fibrotic gene expression in LX2 cells. (**A**) K206R mutation reversed the pyruvate-induced suppression of PKM2 pyruvate kinase activity. LX2 cells were transfected with Flag-PKM2-WT or Flag-PKM2-K206R plasmids for 72 h and then treated with or without sodium pyruvate (5 mM) for 12 h. Pyruvate kinase activity in cell lysates were examined by test kits. (**B**) LX2 cells expressing Flag-PKM2-WT or Flag-PKM2-K206R (72 h post transfection) were treated with or without sodium pyruvate (5 mM, 12 h), followed by DSS cross-linking. Oligomeric states of PKM2 (tetramer: 232 kD, dimer: 116 kD, monomer: 58 kD) were analyzed by Western blot. (**C**) Pyruvate treatment significantly increased the nuclear PKM2 levels in Flag-PKM2-WT-transfected cells. Cytosolic and nuclear proteins were isolated from LX2 cells separately for PKM2 level analysis. (**D**) The K206R mutation blunted the induction of pro-fibrotic gene expression induced by pyruvate. LX2 cells were transfected with Flag-PKM2-WT or Flag-PKM2-K206R for 72 h and treated with or without sodium pyruvate (5 mM, 12 h). Pro-fibrotic gene expression (COL1A1, TIMP1) and protein lactylation levels were assessed by Western blot. (**E**) Ribbon diagram of human PKM2 tertiary structure (PDB entry 6B6U). The figure was generated using the molecular viewer tool PyMol. (**F**) Evolutionary conservation of PKM2 K206 in mammals. Sequence alignment of PKM2 across species (Pan troglodytes, Mus musculus, Rattus norvegicus, Oryctolagus cuniculus, Gallus gallus, Pongo abelii, Bos taurus) revealed a conserved lysine residue (red) corresponding to human K206, critical for lactylation-mediated structural regulation. Data are expressed as the mean ± SD of three sample replicates. *** *p* < 0.001 and **** *p* < 0.0001.

To further investigate the effect of PKM2 K206 lactylation on pyruvate induced pro-fibrotic gene expression, we first evaluated the effect of the PKM2 K206R mutant on PK activity. We found that pyruvate treatment markedly suppressed enzymatic activity in cells transfected with the PKM2-WT plasmid, but had no significant effect in those cells transfected with the PKM2-K206R mutant plasmid (Figure 4A). DSS cross-linking analysis further showed that pyruvate increased the dimeric PKM2 levels (116 kDa) in PKM2-WT-transfected cells but not in cells with the PKM2-K206R mutant (Figure 4B). Additionally, nuclear PKM2 was significantly increased with pyruvate treatment in PKM2-WT-transfected cells, but not in the PKM2-K206R-transfected cells (Figure 4C). These data indicate that the K206 site is critical for PKM2 function, and that pyruvate inhibits PKM2 enzymatic activity partially through increased PKM2 lactylation at the K206 site. Subsequently, we confirmed again that pyruvate treatment (WT-Pyr) robustly upregulated the expression of COL1A1 and TIMP1 and protein lactylation in PKM2-WT-transfected cells. In contrast, PKM2-K206R significantly attenuated pyruvate-induced profibrotic gene expression (Figure 4D). These findings collectively demonstrate that pyruvate promotes PKM2 lactylation at K206, which inhibits PK activity and enhances profibrotic gene expression.

To structurally analyze the effect of the K206 site on PKM2 enzyme activity, we obtained the tertiary structure of PKM2 (PDB entry 6B6U) and observed that the K206 residue was localized within the protein’s B domain (Figure 4E) and adjacent to a critical, enzyme-active site, N75. Evolutionary conservation analysis further revealed that K206 is strictly conserved across mammals, including Pan troglodytes, and extends to avian species such as Gallus gallus (Figure 4F). These findings support the hypothesis that the K206 residue serves as a potential regulatory site for PKM2 function.

## 4. Discussion

**Figure 5:**
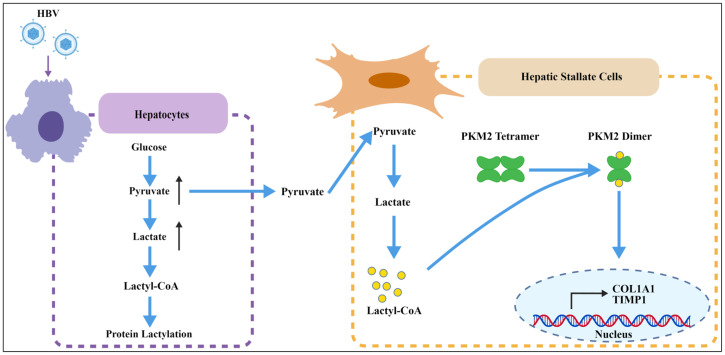
Proposed model for HBV-induced pyruvate promoting liver fibrosis via PKM2 lactylation. HBV infection increases pyruvate in hepatocytes. Elevated pyruvate induces the lactylation of PKM2 in hepatic stellate cells. PKM2 lactylation facilitates dimer formation, thereby promoting the expression of profibrotic genes (COL1A1 and TIMP1).

Liver fibrosis, a core pathological process in chronic HBV infection, is characterized by HSC activation and excessive extracellular matrix deposition in the liver [3,4,5,6]. PKM2, which is highly expressed in activated HSCs, promotes HSC activation through reducing the transformation from dimers with low PK activity to tetramers with high PK activity [27,32]. In this study, we demonstrated that pyruvate, a metabolic intermediate induced by HBV infection, enhanced PKM2 lactylation at K206 site in HSCs, which suppressed its PK activity through stabilizing its dimeric conformation, thereby stimulating profibrotic gene expression, leading to fibrosis (Figure 5).

Metabolic reprogramming is a critical hallmark of malignant cells, and emerging evidence indicates that it also plays a pivotal role in HBV-induced liver fibrosis. HBV infection promotes liver fibrogenesis by altering the liver microenvironment, particularly through increasing aerobic glycolysis [33]. Results from previous studies indicated that the HBV-encoded HBx protein significantly upregulated the expression of key glycolytic enzymes, including hexokinase 2 (HK2) and LDH, in hepatocytes, which resulted in the abnormal accumulation of metabolic intermediates within the liver microenvironment [10,11,34,35]. Consistent with this, our previous [12,13] and current studies showed that HBV infection significantly increased the sera pyruvate and lactate levels in CHB patients and HBV-Tg mice compared with their uninfected counterparts. In 2019, Zhang et al. revealed that lactate, in addition to serving as an energy substrate, could induce lysine lactylation modification [15]. Lactylation modification broadly impacts physiological and pathological processes such as immune regulation, tumor progression, neural activity, and tissue regeneration [36,37,38,39,40]. The role of protein lactylation in HBV-related liver fibrosis remains unclear. In this study, we observed that protein lactylation levels were significantly elevated in liver tissues from HBV-Tg mice compared to the WT controls. Rho et al. discovered that H3K18 lactylation promotes the activation of HSCs and the expression of fibrotic genes [17]. Our previous study found that HBV infection enhanced pyruvate production, which promoted pro-fibrotic gene expression by suppressing PPARα expression and increasing ROS generation [13]. In this study, we confirmed that pyruvate treatment significantly increased profibrotic gene expression, and we also observed that pyruvate treatment enhanced the global protein lactylation levels in HSCs and hepatocytes in a dose-dependent manner (Figure 1D,E). Although we treated the cells with pyruvate, it is well-established that pyruvate is rapidly converted to lactate by lactate dehydrogenase (LDH) under standard culture conditions. In our previous study, we demonstrated that pyruvate treatment significantly increased the lactate levels in the supernatant of cells [13]. Thus, we assumed that pyruvate effectively elevates the intracellular lactate pool, which subsequently drives lactylation. PKM2 is one of the rate-limit enzymes in glycolysis and has been reported to play significant roles in the progression of liver fibrosis [41]. Consistent with these findings, we found that PKM2 knockdown markedly suppressed the expression of pro-fibrotic genes in HSCs. Moreover, PKM2 knockdown also blocked the induction of pro-fibrotic gene expressions by pyruvate, indicating that PKM2 is involved in pyruvate-mediated liver fibrogenesis. Meanwhile, when we examined the pyruvate-induced protein lactylation, we noticed a marked decrease at approximate 58 kDa in PKM2-knockdown cells (Figure 2D). As PKM2 has a molecular weight of ~58 kDa, we hypothesized that pyruvate induced PKM2 lactylation. Multiple studies have demonstrated that the functional diversity and allosteric regulation of PKM2 are highly dependent on PTMs [28]. For instance, the phosphorylation of PKM2 at Y105 disrupts its binding to fructose-1,6-bisphosphate (FBP), leading to dissociation of the enzymatically active tetramer into low-activity dimers. This structural transition facilitates PKM2 nuclear translocation, thereby promoting aerobic glycolysis and tumor growth [42]. Under glucose starvation conditions, Parkin ubiquitinates PKM2 at lysine residues K186 and K206, markedly suppressing PK activity and subsequently inhibiting tumor growth [43]. Wang et al. found that in macrophages, lactate increases the lactylation modification level of PKM2 at the K62 site, inhibits its transition from a tetramer to dimer, suppresses aerobic glycolysis, and promotes the shift of pro-inflammatory macrophages toward a reparative phenotype [29]. In our study, using immunoprecipitation and site-directed mutagenesis, we identified that pyruvate increased PKM2 lactylation at K206. Here, we found that lactylation at K206 promoted PKM2 dimer formation, therefore inhibiting its pyruvate kinase activity and thereby enhancing pro-fibrotic gene expression in HSCs. In contrast to the findings by Wang et al., we found that mutation of the K62 site did not significantly alter the PKM2 lactylation levels induced by pyruvate. These results indicate that the lactylation modification of PKM2 may occurred at different sites in different cells or induced by different substrates, reflecting the complexity of post-translational regulation of PKM2. The complex interaction network between hepatocytes and hepatic stellate cells is of great significance in liver fibrosis. In our study, global hepatic proteins of HBV-Tg mice were found to undergo lactylation (Figure 1C), and global protein lactylation was induced by pyruvate treatment in both the NTCP-HepG2 and LX2 cells (Figure 1D,E). We previously reported that HBV infection induced pyruvate and lactate production in the supernatant of hepatocytes, which exerted an effect on pro-fibrotic gene expression in both hepatocytes and hepatic stellate cells [13]. Additionally, in LX2 cells, we also observed that PKM2 lactylation was elevated after exposure to pyruvate or conditioned medium from HBV-infected hepatocytes, suggesting an indirect paracrine effect. Moreover, others have reported that TGF-β and PDGF were elevated in HBV-related fibrosis [44,45]. However, it remains unclear whether PKM2 lactylation occurs in hepatocytes and whether it promotes the secretion of these cytokines. Further investigation into the detailed mechanisms is warranted.

Certainly, this study has certain limitations. Firstly, we were not able to construct all 16 potential lysine mutants of PKM2, and lactylation may also occur at the 6 remaining mutant lysine sites that we did not clone successfully. Secondly, while our data strongly suggest a link between PKM2 K206 lactylation and fibrotic response, causal in vivo evidence is currently lacking. Future studies exploring the complex interplays among HBV-induced pyruvate production, PKM2 lactylation modification, and liver fibrosis progression using conditional PKM2 K206R knock-in mice in HBV background are warranted.

In summary, our current study demonstrates that HBV-induced pyruvate increases profibrotic gene expression through enhanced PKM2 lactylation at K206, which subsequently promotes the formation of PKM2 dimers, leading to decreased pyruvate kinase activity. These findings may provide novel therapeutic targets for developing drugs to mitigate or even reverse HBV-related liver fibrosis.

## 5. Conclusions

In summary, our study indicates the critical role of PKM2 lactylation in liver fibrosis. We demonstrated that HBV infection increases pyruvate production, which promotes PKM2 lactylation at lysine 206 in HSCs. This modification thereby facilitates the formation of PKM2 dimers and attenuates its enzymatic activity, ultimately stimulating the expression of profibrogenic genes in HSCs.

## Figures and Tables

**Table 1:** Primer sequences used for RT-qPCR.

Gene Name	Forward (5′-3′)	Reverse (5′-3′)
*β-actin*	GATGAGATTGGCATGGCTTT	GTCACCTTCACCGTTCCAGT
*PKM2*	ACGAGAACATCCTGTGGCTG	GCTCGACCCCAAACTTCAGA
*COL1A1*	AACATGACCAAAAACCAAAAGTG	CATTGTTTCCTGTGTCTTCTGG
*TIMP1*	GAGAGACACCAGAGAACCCAC	CCACAGCAACAACAGGATGC

**Table 2:** Baseline characteristics of the patients.

	Non-HBV (n = 7)	CHB-I (n = 28)	CHB-F (n = 27)	*p* Value			Effect Size			Power		
				P1	P2	P3	P1	P2	P3	P1	P2	P3
Age (years)	46.0 (30.0–50.0)	36.0 (30.5–42.5)	48.0 (43.0–51.0)	0.180	0.442	0.0001	1.09	0.13	1.15	0.71	0.06	0.99
Male (n, %)	4 (57.1)	13 (46.4)	17 (63.0)	0.617	0.781	0.223	0.09	0.05	0.17	0.08	0.06	0.24
ALT (U/L)	23.0 (17.0–29.0)	39.0 (27.5–121.3)	24.0 (16.0–33.0)	0.006	0.798	0.0002	0.93	0.32	0.78	0.55	0.11	0.79
AST (U/L)	21.0 (19.0–24.0)	29.0 (22.0–62.0)	23.0 (20.0–26.0)	0.018	0.442	0.005	0.98	0.36	0.80	0.60	0.13	0.43
TBIL (μmol/L)	13.7 (10.4–28.6)	16.2 (12.5–19.1)	12.7 (10.7–16.0)	0.805	0.565	0.077	0.14	0.45	0.39	0.06	0.17	0.28
Lactate (μmol/mL)	2.1 (1.6–2.3)	5.8 (4.2–9.1)	9.8 (6.9–12.2)	0.0064	<0.0001	0.0046	2.35	3.29	1.07	1.00	1.00	0.97

Data are presented as the median (25th–75th percentile). Abbreviations: ALT, alanine aminotransferase; AST, aspartate aminotransferase; TBIL, total bilirubin; CHB-I, inactive carrier stage of chronic hepatitis B virus; CHB-F, advanced fibrotic stage of chronic hepatitis B virus. P1: compared between Non-HBV and CHB-I groups; P2: compared between Non-HBV and CHB-F groups; P3: compared between CHB-I and CHB-F groups.

## Data Availability

The original contributions presented in this study are included in the article. Further inquiries can be directed to the corresponding authors.
